# Pituitary dysfunction in granulomatosis with polyangiitis

**DOI:** 10.1007/s11102-017-0811-0

**Published:** 2017-05-24

**Authors:** Daniela Esposito, Penelope Trimpou, Dario Giugliano, Mats Dehlin, Oskar Ragnarsson

**Affiliations:** 1 Department of Endocrinology, Institute of Medicine, Sahlgrenska Academy, University of Gothenburg, Gothenburg, Sweden; 2 Sahlgrenska University Hospital, Gröna Stråket 8, 413 45 Gothenburg, Sweden; 3 Department of Medical, Surgical, Neurological, Metabolic Sciences and Aging, University of Campania “Luigi Vanvitelli”, Naples, Italy; 4 Department of Rheumatology, Sahlgrenska University Hospital, 413 45 Gothenburg, Sweden

**Keywords:** Pituitary insufficiency, Diabetes insipidus, Cyclophosphamide, Vasculitis, Wegener´s granulomatosis

## Abstract

**Purpose:**

Granulomatosis with polyangiitis (GPA) is a multisystem disease, characterized by necrotizing small-vessel vasculitis, which mainly affects the respiratory tract and the kidneys. Pituitary involvement in GPA is rare, present in about 1% of all cases of GPA. To date, only case reports or small case series have been published. Herein we report clinical features, imaging findings, treatment and outcomes in three patients with GPA-related pituitary dysfunction (PD).

**Methods:**

A retrospective analysis of three cases of GPA-related PD was conducted, followed by systematic review of the English medical literature using PubMed.

**Results:**

The three cases include three women aged between 32 and 37 years. PD was the presenting feature in one and two developed PD in the course of the disease. All patients had a pituitary lesion on MRI. Conventional treatment with high doses of glucocorticoids and cyclophosphamide led to resolution or improvement of the MRI abnormalities, whereas it was not effective in restoring PD. A systematic review identified 51 additional patients, showing that GPA can lead to partial or global PD, either at onset or, during the course of the disease. Secondary hypogonadism is the predominant manifestation, followed by diabetes insipidus (DI). Sellar mass with central cystic lesion is the most frequent radiological finding.

**Conclusion:**

GPA should be carefully considered in patients with a sellar mass and unusual clinical presentation with DI and systemic disease. Although conventional induction-remission treatment improves systemic symptoms and radiological pituitary abnormalities, hormonal deficiencies persist in most of the patients. Therefore, follow-up should include both imaging and pituitary function assessment.

## Introduction

Granulomatosis with polyangiitis (GPA), previously known as Wegener’s granulomatosis, is a rare disease of unknown aetiology. GPA is characterized by necrotizing small-vessel vasculitis, which mainly affects the upper respiratory tract, lungs and kidneys [1, 2]. However, GPA can potentially involve any organ or tissue, including skin, eye, trachea, nervous system, gingiva, breast and prostate.

Pituitary gland involvement in GPA is rare and can lead to partial or global pituitary dysfunction (PD), either at onset or in the course of the disease [3]. The first case was described in 1953 by Ahlstrom et al. [4]. Since then only case reports [5–10] or small case series [3, 11] have been published in the English medical literature and long-term outcomes have rarely been reported.

The aim of the current report is to describe the clinical features of PD in three patients with GPA, and to systematically review clinical features, imaging findings, treatment and outcome.

## Case 1

A 37-year-old woman was in excellent health until two months before admission to the emergency department where she reported recurrent obstructive sinusitis followed by progressive dyspnoea, fever, fatigue and headache. On admission, a granuloma in the nasal mucosa, without discharge or bleeding, was detected. Physical examination was otherwise unremarkable, including vital signs, auscultation of the heart and lungs, and oxygen saturation. The haemoglobin level was 117 g/L, platelet count 578 × 10^9^ and c-reactive protein was 240 mg/L (normal; <5). White blood cell count was 17.6 × 10^9^ and neutrophils 8.3 × 10^9^. Electrolytes, creatinine, liver tests, glucose, myoglobin, creatine kinase, TSH and free thyroxin in serum were within the normal reference range. Computed tomography (CT) of the head showed thickening of the maxillary sinus and nasal mucosa.

Cytoplasmic antineutrophil cytoplasmic antibodies (c-ANCA) were positive (titer >20) and anti-proteinase 3 was 27 IU/mL (normal <5). The granuloma of the nasal mucosa was removed surgically. Histological examination revealed necrotizing vasculitis, granulomatous inflammation and giant cells, consistent with the diagnosis of GPA. Thereafter, prednisolone and methotrexate were administered with good effect.

**Fig. 1:**
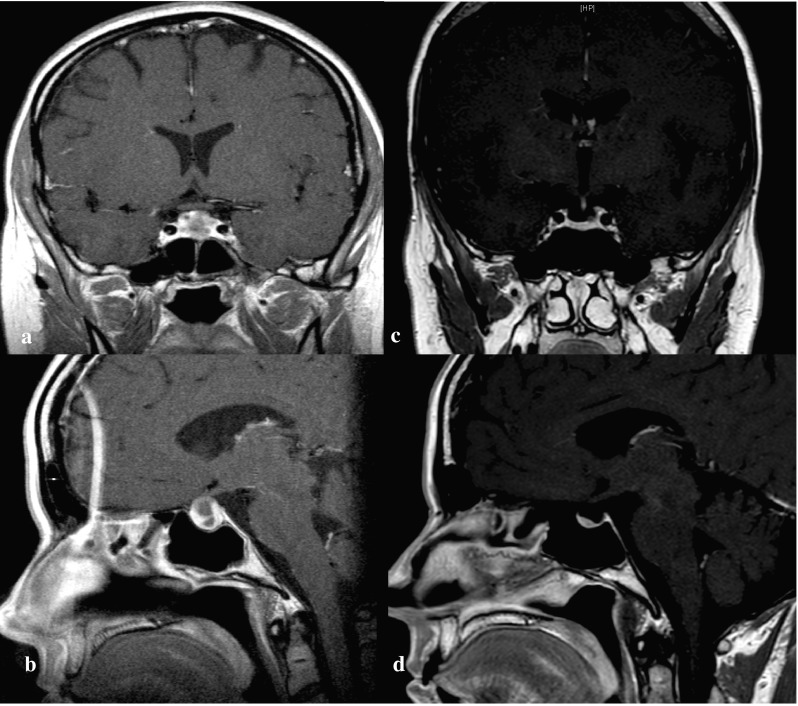
T1 weighted MRI (coronal and sagittal view) of a 37-year-old woman with GPA (Case 1) showing a sellar mass with peripheral enhancement and central cystic lesion at onset (**a** and **b**) and 8 years after treatment showing a partial empty sella (**c** and **d**)

During the next 2 months, the patient developed progressive polydipsia and polyuria, with an estimated 24-h urine output of 12 l. GPA-related glomerulonephritis was ruled out and the patient was referred for endocrine evaluation due to clinical suspicion of diabetes insipidus (DI). A water deprivation test was performed. After 6 h, the patient had lost 2.6 kg of her body weight (3.75%) and the test was interrupted. Urine osmolality increased only marginally during the test, from 91 mosmol/kg at baseline, to 121 mosmol/kg upon termination. Serum sodium increased from 142 to 150 mmol/L. Following administration of intranasal desmopressin (4 µg), urine osmolality increased to 379 mosmol/kg, confirming the diagnosis of central DI. Intranasal desmopressin 10 µg twice daily normalised the urine output and serum sodium levels. Further hormonal assessment revealed normal anterior pituitary function. MRI of the pituitary gland revealed a sellar mass extending into the suprasellar cistern with peripheral enhancement and central cystic lesion (Fig. 1a, b). The stalk was deviated to the right and the optic chiasm was slightly compressed. Perimetry revealed, however, no visual field defects. Due to PD, the cytotoxic agent was switched to cyclophosphamide 1.5 g once a month for nine consecutive months. After 6 months, imaging showed reduction of the pituitary mass.

Two years later, the patient reported fatigue and weakness accompanied by profuse sweating, along with amenorrhea throughout the last 3 months. The hormonal work-up revealed low serum levels of estradiol, FSH and LH, consistent with secondary hypogonadism, and estrogenic therapy was prescribed. Moreover, IGF-1 was 86 (normal range 68–225 µg/l) and insulin tolerance test (ITT) confirmed severe growth hormone (GH) deficiency. GH replacement therapy was started with significant symptomatic improvement. Pituitary MRI demonstrated a further reduction of the pituitary mass and a partial empty sella (Fig. 1c, d). For 9 years the patient has been regularly followed at our clinic and has remained stable on replacement therapy with desmopressin, GH and oestrogen, while on a maintenance dose of methotrexate.

## Case 2

**Fig. 2:**
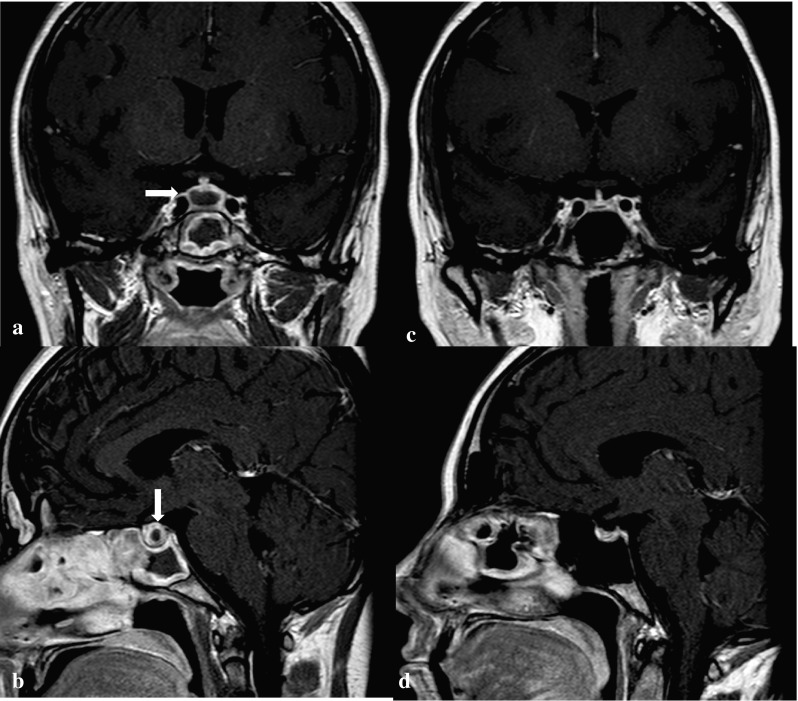
T1-weighted MRI (coronal and sagittal view) of a 39-year-old woman (Case 2) showing a sellar mass with central cystic lesion before (**a** and **b**) and after (**c** and **d**) conventional treatment for GPA associated PD

A 36-year-old woman presented with cough, fever and severe myalgia at the emergency department at our hospital. She had been in good health until 4 months before the admission when excessive thirst, polydipsia and polyuria developed. After a diagnostic work-up at another hospital, central DI was diagnosed. The anterior pituitary function was normal. Desmopressin acetate nasal spray was prescribed with good effect. On MRI a cystic pituitary mass was identified (Fig. 2a, b) and surgical extirpation was scheduled. Few weeks later, before any surgical procedure, otitis media with tympanic membrane perforation was diagnosed and treated with antibiotics, without clinical improvement.

On admission, the patient reported successively increased problems with myalgia, lethargy, non-productive cough and episodic fever accompanied by profuse sweating for the last 3 weeks. On physical examination she was pale and appeared distressed due to generalized and severe muscle pain. The vital signs and oxygen saturation were normal. Auscultation of the heart and lungs was normal. The left tympanic membrane was perforated and serous fluid was observed in the external auditory canal. The haemoglobin level was 106 g/L, platelet count 407 × 10^9^ and c-reactive protein 120 mg/L. White blood cell count, electrolytes, creatinine, liver tests, glucose, myoglobin, creatine kinase, TSH, free thyroxin and cortisol in serum were within the normal reference range. Chest X-ray showed an infiltrate in the upper part of the left lower lobe. A preliminary diagnosis of pneumonia was made and intravenous fluids and antibiotics were administrated.

**Fig. 3:**
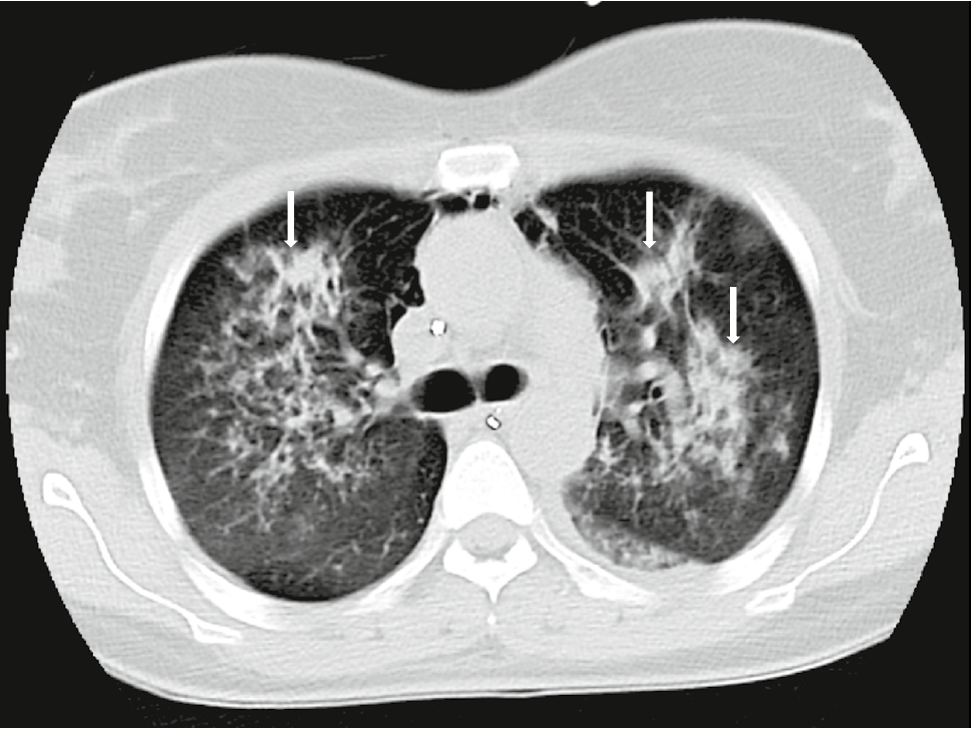
CT scan showing extensive bilateral consolidations in the lungs (Case 2)

During the next 3 days, her clinical status gradually deteriorated. The respiratory rate increased from 12 to 28 breaths per minute, oxygen saturation decreased from 98 to 92% and pCO2 increased from 4.4 to 6.9 kPa. CT scan showed extensive bilateral consolidations in the lungs (Fig. 3). Despite no previous haemoptysis, fresh blood was seen in both main bronchi at bronchoscopy. Subsequently, respiratory failure developed and the patient was intubated and mechanical ventilation was started.

Due to the clinical picture with bilateral pulmonary haemorrhage and otitis media, GPA was strongly suspected. c-ANCA was positive (titer >160, normal <20) and anti-proteinase three level was 1400 IU/mL (normal <5). Anti-myeloperoxidase and anti-glomerular basement membrane antibodies were negative. Microscopic examination of the urine showed 11–30 erythrocytes per high power field (×400), erythrocyte sedimentation rate was 111 mm and creatinine was 45 μmol/L (normal 45–90). Treatment with methylprednisolone (1 g daily for 3 days) and five sessions of plasmapheresis per day were started. In addition, 1.5 g of cyclophosphamide was administrated with clinical improvement.

Long-term therapy with immunosuppression resulted in overall clinical improvement. Further MR examination showed a normal-size gland and normal pituitary signal characteristics, with the exception of persistent loss of the posterior lobe T1 hyperintensity (Fig. 2c, d). Despite adequate response of the systemic disease to the treatment and resolution of imaging findings, DI persisted and the patient remained stable on the same desmopressin therapy with normal urine output and serum sodium levels.

## Case 3

A 32 year-old woman was referred to the endocrine clinic for evaluation of polydipsia and polyuria. GPA had been diagnosed 5 months earlier due to recurrent sinusitis and otitis media, accompanied by headache and hearing loss, not responding to antibiotic treatment. On physical examination, a granuloma had been detected in the left middle ear and was surgically removed. c-ANCA was positive (titer >80, normal <20) and anti-proteinase 3 was 93 IU/mL (normal; <5). Thus, the clinical and biochemical findings were consistent with diagnosis of GPA and treatment with azathioprine and high dose of corticosteroids was started. The patient responded promptly and achieved remission.

**Fig. 4:**
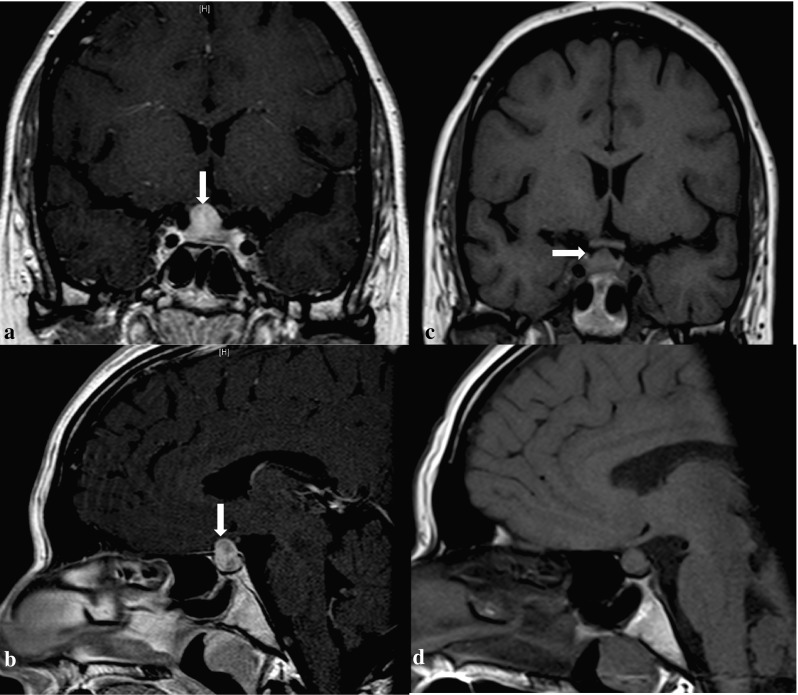
T1-weighted MRI (coronal and sagittal view) of a of 32-year-old woman (Case 3) with GPA associated PD showing a sellar mass with homogenous enhancement before (**a** and **b**) and after (**c** and **d**) treatment

Because of progressive polydipsia and polyuria for 12 weeks she was admitted to the endocrine clinic. She was in good general condition. DI was diagnosed based on polydipsia, polyuria, low urine osmolality and elevated serum osmolality. The anterior pituitary function was normal. Secondary adrenal insufficiency was not investigated, since the patient was already on corticosteroid treatment. The patient received treatment with desmopressin with good effect. A pituitary MRI revealed a sellar mass with homogeneous enhancement, thickening of the pituitary stalk and mild compression of optic chiasm (Fig. 4a, b). Visual field acuity test did not reveal any significant defects.

A remission-induction treatment for GPA-related PD with cyclophosphamide was administered. 3 months later, imaging showed a mild regression of the pituitary mass. One year later a marked improvement was seen, with no compression of the optic chiasm (Fig. 4c, d). Repeated MRI and clinical controls during the last 3 years have indicated a stable disease and normal anterior pituitary function on a maintenance dose of azathioprine and desmopressin.

## Discussion

GPA is a rare autoimmune disease characterized by necrotising granulomatous vasculitis that mainly involves the upper and the lower respiratory tracts and the kidneys, although any organ may be affected [1, 12]. One-third of patients with GPA have a neurological engagement, primarily in the form of peripheral neuropathy due to small-vessel vasculitis. Central nervous system involvement occurs in 7–18% of all cases [13]. First described in 1953 [4], PD in GPA is rare and present in about 1% of all GPA patients [3]. In our catching area (western Sweden) approximately 250 to 300 patients have GPA. To our knowledge, of those, only the patients presented in the current report have developed PD, i.e. the prevalence is in agreement with previous estimates [3].

Inflammatory and granulomatous lesions in the pituitary gland are rare causes of PD [14, 15]. Of these, lymphocytic hypophysitis is the most common. Pituitary involvement in GPA is a form of a so called “secondary hypophysitis”, a term first introduced for more than 20 years ago to indicate a pituitary inflammation resulting from systemic diseases or pituitary near-lying lesions [16]. Other causes of granulomatous pituitary lesions are sarcoidosis, tuberculosis, idiopathic giant cell granulomatosis, Crohn´s disease and Takayasu´s vasculitis [14]. To date, only two case series and 34 cases have been published describing GPA-related PD, resulting in 51 patients reported in the English medical literature.

### Pathophysiology

The pathophysiology behind PD in GPA is not entirely known. Three main pathogenic mechanisms are thought to be involved [5, 17]. First, and the most commonly implicated mechanism, is a direct intracranial extension of the granulomatous process from the nasal or paranasal granuloma to the pituitary gland. The second one is a vasculitis of the pituitary vessels that results in ischemic and/or hemorrhagic infarctions. The third possible mechanism is a development of new granulomas in the pituitary gland itself.

### Clinical features

Clinical features of PD are rarely present at onset of GPA. In fact, in a recent case series, only one of nine patients had PD as a presenting symptom [11]. Instead, the diagnosis of PD more commonly occurs subsequently, between 2 months to 15 years after the diagnosis of GPA. Worth to note is that the symptoms of PD in GPA may be nonspecific including weakness, headache, or fatigue, which may lead to underdiagnosing or a delay in the diagnosis.

The three cases reported here, all women aged between 32 and 37 years, share a similar clinical profile with patients previously reported in the literature (Table 1). PD was the presenting feature in one (Case 2) and two developed PD in the course of the disease (Case 1 and 3). Yong et al. reviewed 22 cases published in the English medical literature between 1966 and 2006 [18]. The mean age at diagnosis of PD in GPA was 38 years (range 13–71) and 74% of patients were female. Two recent case series described patients with a somewhat different profile, though. In the French Vasculitis Study Group cohort the mean age at the onset of PD in nine patients with GPA was 51 years (range 24–77), five were women and four men [11]. Kapoor et al. described eight cases with a mean age of 48 years (range 28–68), with a 1:1 male to female ratio [3].

**Table 1:** Demographic, clinical and hormonal features in patients with GPA related pituitary dysfunction

Case	Age	Sex	Active disease at other sites			Pituitary dysfunction							
			ENT	Lung	Renal	ANCA	PR3	DI	FSH/LH	TSH	ACTH	GH	PRL
1	37	F	+	−	−	Pos	Pos	+	+	−	CT	+	−
2	36	F	+	+	−	Pos	Pos	+	−	−	CT	−	−
3	32	F	+	−	−	Pos	Pos	+	−	−	CT	−	−

*ENT* involvement of upper respiratory tract (ear, nose and throat), *CT* under corticosteroids, therefore ACTH and cortisol assessment is not indicated

One or more pituitary axes functions can be affected in PD caused by GPA. Consequently, every pituitary hormone function should be investigated. One of the most common clinical entities is DI, isolated or associated with anterior PD [6–10]. Conversely, anterior PD, without coexisting DI is uncommon. De Parisot et al. recently reported that secondary hypogonadism is the most common endocrinopathy in pituitary GPA, closely followed by DI (detected in 78 and 71% of all cases, respectively) [11]. This was in agreement with a previous case series, showing hypogonadism in 88% and DI in 75% [3]. The pathogenic mechanism behind hypogonadism remains unclear and is presumably multifactorial. Several factors can affect the hypothalamic-pituitary-gonadal axis, including hyperprolactinemia, the chronic disease itself and pharmacologic treatment. It is well known that treatment with both glucocorticoids and cyclophosphamide can provoke hypothalamic-pituitary-gonadal axis suppression [19]. The high rate of hypogonadism due to GPA itself may therefore be overestimated. It is unclear whether hypogonadism reported in the previous case series was diagnosed at onset of GPA or developed after the treatment was initiated. All our patients had DI and only one developed hypogonadism (Case 1) and we cannot exclude that it was related to treatment with high dose of steroids and cyclophosphamide.

Other hormone deficiencies in GPA-related pituitary dysfunction are not as common as DI and hypogonadism. Low levels of free thyroxine, consistent with diagnosis of central hypothyroidism, have been reported in 54% of patients. Secondary adrenal insufficiency occurs in 39% of cases and growth hormone deficiency (GHD) in 20% [11]. Finally, hyperprolactinemia is reported in 37% of patients, probably due to pituitary stalk compression. The prevalence of GHD in GPA-associated PD is probably underestimated. In fact, the diagnosis of GHD in the previous reported cases was based on abnormally low IGF-1 and not on dynamic testing. For the first time we report a patient who was diagnosed with GHD through an insulin tolerance test, the gold standard for diagnosing GHD [20]. GHD in this case developed 2 years after being diagnosed with GPA despite remission of the systematic disease. In granulomatous pituitary lesions of other causes than GPA, GHD is reported to be the most common endocrinopathy followed by hypogonadism, central hypothyroidism and secondary adrenal insufficiency [14]. On the contrary, the prevalence of GHD in pituitary GPA is reported to be seen in only 20% of the patients. However, none of the patients described in the medical literature had any dynamic testing. Therefore, we recommend performing dynamic stimulation tests for evaluating GHD, if clinical and biochemical findings are suspicious for this hormone deficit.

Visual field test is an important tool in order to complete the diagnostic work-up in pituitary GPA. Thus, all patients with a sellar mass should undergo visual field evaluation. Kapoor et al. [3] disclosed visual deficits in 40% of their patients, compared to 17% reported in previous literature [13]. In contrast, none of our patients presented with abnormalities on visual field testing.

### Radiological findings

PD is frequently diagnosed on the basis of head imaging, commonly performed to investigate headache and/or sinusitis in patients with GPA. The most frequent finding on MRI in patients with pituitary GPA is a sellar mass with peripheral enhancement and a central cystic hypointense lesion. Absence of the posterior hyperintense signal on T1-weighed images and a thickening of the pituitary stalk are also common [18, 21]. The most frequent pituitary diseases in general are pituitary adenomas. Although pituitary adenomas are usually isointense on MRI, some have a similar appearance as patients with GPA, i.e. peripheral enhancement around a hypointense lesion. However, pituitary adenomas only very rarely present with DI and other diseases such as GPA, hypophysitis, sarcoidosis, Langerhans cell histiocytosis and metastasis should be carefully considered in patients with new onset polyuria and polydipsia [14].

Two of our patients (Case 1 and 2) presented with a typical pituitary cystic lesion with peripheral enhancement. One (Case 3) had a sellar mass with homogeneous contrast enhancement. The correct diagnosis of pituitary lesions is essential to avoid unnecessary biopsy or surgical treatment, which is not primarily recommended in pituitary GPA, given the excellent response to treatment with immunosuppressive therapy.

### Treatment and outcome

Conventional remission-induction treatment for PD in GPA consists of high-dose glucocorticoids combined with oral or intravenous cyclophosphamide, with a reported remission in two-third of patients [5, 11]. De Parisot et al. reviewed 51 cases in English and French medical literature, showing that 69% of all patients were treated with conventional treatment. After a median follow-up of 5 years, 11% had a relapse of systemic disease [11]. Induction treatment without cyclophosphamide is associated with a relapse in 50% of the patients after a median follow-up of 4.5 months [5]. Thus, cyclophosphamide seems to be more effective in pituitary GPA than other immunosuppressive treatments. Patients that are refractory to the conventional therapy can be successfully treated with rituximab, approved in severe ANCA-associated vasculitis [22]. Recent trials have shown that GPA patients treated with rituximab achieve complete remission more frequently than those who are treated with cyclophosphamide [23]. These findings suggest that clinicians may consider the use of rituximab as first-line therapy [24]. However, treatment experience with rituximab for PD in GPA is limited and further studies are needed [3, 5].

All our patients were treated with glucocorticoids and cyclophosphamide. Two patients had a complete resolution of the pituitary mass (Case 1 and 2) and one had a partial resolution. However, all our patients had a persistent PD. It has been reported that 65% of all cases have a complete or partial resolution of the pituitary lesions on MRI assessment, after the treatment [11]. Interestingly, there is no relationship between hormonal, radiologic and systemic outcomes, thus follow-up should include both imaging and pituitary function assessment. Despite a high rate of systemic disease remission and regression of radiological abnormalities, the outcome of pituitary function is less favorable. In fact, De Parisot et al. reported that 86% of the patients have permanent deficiency of one or more of the pituitary hormones, probably due to a permanent pituitary damage related to necrotizing granulomatous inflammation of the gland [11].

No data are available concerning long-term outcome in pituitary GPA. Patients with hypopituitarism, irrespective of underlying aetiology, have increased morbidity and mortality rates compared to the general population [25, 26]. Whether patients presenting with PD in the context of GPA have a more aggressive disease burden per se, and to what extend this could affect the overall prognosis in these patients is unknown.

## Conclusion

GPA should be carefully considered in patients with a sellar mass and unusual clinical presentation with DI and systemic disease, mainly affecting the respiratory tract and kidneys. GPA can lead to partial or global pituitary dysfunction in around 1% of cases. It is important that clinicians are aware of the potential for pituitary involvement in this rare disease in order to avoid unnecessary biopsy or surgical treatment of sellar lesions. Although conventional induction-remission treatment (GCs and cyclophosphamide) improves systemic symptoms and radiological pituitary abnormalities, hormonal deficiencies persist in most of the patients and can occur even throughout or after the treatment. Therefore, follow-up should include both imaging and pituitary function assessment, including dynamic tests, when appropriate.
